# Adoption of climate-smart agricultural practices among smallholder farmers in Western Kenya: do socioeconomic, institutional, and biophysical factors matter?

**DOI:** 10.1016/j.heliyon.2021.e08677

**Published:** 2021-12-25

**Authors:** Collins M. Musafiri, Milka Kiboi, Joseph Macharia, Onesmus K. Ng'etich, David K. Kosgei, Betty Mulianga, Michael Okoti, Felix K. Ngetich

**Affiliations:** a Department of Land and Water Management, University of Embu, PO BOX. 6-60100, Embu, Kenya; b Cortile Scientific Limited, PO BOX 34991 – 00100, Nairobi, Kenya; c Kenyatta University, Department of Geography, PO Box 43844-00100, Nairobi, Kenya; d University of Embu, Department of Agricultural Resource Management, PO Box 6-60100, Embu, Kenya; e Department of Agricultural Economics and Resource Management, Moi University, PO Box 3900 – 30100, Eldoret, Kenya; f Kenya Agricultural and Livestock Research Organization (KALRO), Sugar Research Institute (SRI), PO Box 44-40100, Kisumu, Kenya; g Kenya Agricultural and Livestock Research Organization (KALRO), Headquarters, PO BOX 30148 – 00100, Nairobi, Kenya; h Jaramogi Oginga Odinga University of Science and Technology (JOOUST), School of Agricultural and Food Sciences, PO Box 210 - 40601, Bondo, Kenya

**Keywords:** Soil fertility decline, Climate-smart agriculture, Climate change, Multivariate probit model, Ordered probit model

## Abstract

Rigorous efforts should be channeled to the current low adoption of climate-smart agricultural practices (CSAPs) in sub-Saharan African countries to improve food production. What determines the adoption level and intensity of CSAPs among smallholder farmers in Kenya? While considering their joint adoption, smallholder farmers' CSAPs adoption determinants were assessed based on a sample size of 300 smallholder farmers in Western Kenya. The CSAPs considered were animal manure, soil water conservation, agroforestry, crop diversification, and crop-livestock integration. A multivariate and ordered probit models were used to assess the determinants of joint adoption of CSAPs in Western Kenya. Both complements and substitutes between CSAPs were established. The multivariate probit analysis revealed that household head's gender, education, age, family size, contact with extension agents, access to weather information, arable land, livestock owned, perceived climate change, infertile soil, and persistent soil erosion influenced CSAPs adoption. The ordered probit model revealed that gender, arable land, livestock owned, soil fertility, and constant soil erosion were crucial determinants of CSAPs adoption. The findings implied that policymakers and relevant stakeholders should consider farmer, institutional, and biophysical factors in upscaling or promoting the adoption of CSAPs.

## 1 Introduction

Climate change is a major significant hurdle to agricultural production globally. The climate change impacts on agricultural production are predominant in developing countries such as sub-Saharan Africa (SSA), where agriculture is rainfed dependent [33, 44]. Climate change manifests as dry spells, meteorological droughts, flooding, unreliable rainfall, cropping calendar changes, and increased atmospheric temperature [5, 32]. Climate change induces crop failure and livestock losses culminating in food insecurity and posing severe threats to society's wellbeing [43]. Despite the climate change impacts in the agricultural sector, producing more food is needed for the increasing population in SSA countries, Kenya included is essential. Therefore, the need for interventions such as adopting climate-smart agricultural practices (CSAPs) among smallholder farmers.

Smallholder farmers are faced with multiple climate change shocks, including floods, erratic rains, dry spells, drought, among others [50, 51]. The climate change shocks significantly affect agricultural productivity, including total crop failure and livestock losses. Therefore, smallholder farmers adopt single or multiple agricultural practices to cope with the impacts of climate change [3, 43, 51]. The CSAPs such as animal manure, soil water conservation, agroforestry, crop diversification, and crop-livestock integration improve food security and community welfare [19, 22, 31, 38, 43]. The above CSAPs were selected based on the literature on using the practices and expert knowledge of the study area. Despite the novel gains from CSAPs in enhancing food security and community wellbeing, their adoption levels remain relatively low [34]. This adoption varies across practices, regions and the rates range from low to high [5, 15, 24, 30]. However, there is limited literature on adopting the combination of agricultural practices such as animal manure, soil water conservation, agroforestry, crop diversification, and crop-livestock integration in Western Kenya to mitigate the impacts of climate change. Therefore, assessing adoption levels and intensity of CSAPs is vital in promoting policy formulation, technology dissemination, and improving livelihoods.

Smallholder farmers adopt CSAPs to cope with climate change shocks. Smallholder farmers are faced with complex decisions either not to adopt or adopt a single or combination of technologies for climate change mitigation and adaptation [30]. The adoption of CSAPs is mainly driven by expected utility Rabin [37], where a farmer could adopt a practice if the pay-off is better than not adopting. However, smallholder farmers are also faced with the decision to adopt a bundle of CSAPs [21, 25]. The adoption of a specific practice could be conditioned to another. Therefore, assessing the determinants of the adoption of CSAPs should test the assumption of the interdependencies between them [35]. Previous studies found interdependencies between practices while estimating determinants of simultaneous adoption of agricultural innovation, thus assuming independence between them could produce biased outcomes [10, 25, 30, 41]. Adopting CSAPs could be influenced by geographical location, farmer demographics, institution traits, biophysical factors, and the practice under consideration. Since smallholders could consider the combination of technologies, exploring the determinants of adoption intensity is equally essential.

Despite the potential of integrating CSAPs to improve food security and community wellbeing, adopting a bundle of practices is limited by various factors such as high initial cost and technical know-how. Therefore, a smallholder farmer could adopt none, single, or several practices based on their ability. Hence, the need to evaluate determinants of CSAPs adoption among smallholder farmers across diverse locations to design pro-farmer policies that could foster intervention adoption and improve food security against the backdrop of changing climate. The determinants of smallholders' joint adoption of CSAPs in Western Kenya were assessed. The research question addressed was: what determines the smallholders' adoption of multiple interrelated CSAPs in Western Kenya?

## 2 Methodology

### 2.1 Study area

The study was conducted in Alego-Usonga and Ugenya sub-counties in Siaya County, Western Kenya. Alego-Usonga and Ugenya sub-counties cover 599 km^2^ and 324 km^2^, respectively, 36.48% of Siaya County. Alego-Usonga and Ugenya are inhabited by 224,343 and 134,354 persons, respectively, 36.11% of the Siaya County population [18]. The study area is located in diverse agro-ecological zones, including Upper midland (UM1) and low midlands (LM1-5) [13].

The elevation ranges from 1140 to 1500 m above sea level. The site experience long-term annual temperature and rainfall ranging from 20.9 to 22.3 °C and 800–2000 mm [13]. The rainfall is bimodally distributed, where long rains occur from March to June and short rains from September to December each year. This results in two full cropping seasons per year. The main economic activity is crop and livestock farming. However, the rainfalls are highly erratic and unpredictable, leading to crop-livestock losses and food insecurity. The main climatic hazards in the study area include dry spells, flooding, and heat stress [51]. The threats significantly affect crop and livestock production. Therefore, smallholder farmers are forced to explore different CSAPs to mitigate the adverse climate change impacts. Most of the smallholder farmers in the area grow orphan crops such as cassava (*Manihot esculenta*), millet (*Panicum miliaceum*), sorghum (*Sorghum bicolor*), cowpea (*Vigna unguiculata*), chickpea (*Cicer arietinum*), and groundnut (*Arachis hypogaea*). They also grow food crops such as common bean (*Phaseolus vulgaris*) and maize (*Zea mays*). The predominant livestock reared includes goat, sheep, cattle, and poultry. Fishing is also a joint economic activity in the study area.

### 2.2 Study variables description

Smallholder farmers were asked to explain their encounters with the changing climate over the last ten years. Following the experience of smallholder farmers with climate change, they were asked to enumerate CSAPs they had adopted. The main CSAPs adopted by smallholder farmers to improve agricultural productivity and cope with climate change included the use of animal manure, agroforestry, soil water conservation, crop diversification, and crop-livestock integration (Table 1). The practices mentioned were consistent with the literature [5, 21, 25, 32, 35]. Five CSAPs were utilized as the outcome variables. The adoption intensity indicates the number of CSAPs adopted by a smallholder farmer (Table 2).

**Table 1:** Climate-smart agricultural practices adopted by smallholder farmers in Western Kenya.

CSA practices	Description	Mean	Std Dev.
Animal manure	Dummy = 1 if the household adopted animal manure, 0 otherwise	0.32	0.27
Soil water conservation	Dummy = 1 if the household adopted soil water conservation, 0 otherwise	0.65	0.48
Agroforestry	Dummy = 1 if the household adopted agroforestry, 0 otherwise	0.30	0.46
Crop diversification	Dummy = 1 if the household adopted crop adjustments, 0 otherwise	0.78	0.42
Crop-livestock integration	Dummy = 1 if the household adopted crop livestock integration, 0 otherwise	0.44	0.30

**Table 2:** Intensity of climate-smart agriculture practices adoption among smallholder farmers in Western Kenya.

Intensity of adoption (Number of technologies)	Frequency	Percentage (%)
0	6	2.00
1	45	15.00
2	105	35.00
3	91	30.33
4	45	15.00
5	8	2.67
**Total**	**300**	**100**

The authors' expertise on the subject matter and available literature on CSAPs adoption were used to select independent and dependent variables [10, 16, 25, 27, 30, 39]. The five CSAPs, animal manure, agroforestry, soil water conservation, crop diversification, and crop-livestock integration, were measured as one (1) if the smallholder farmer adopted a specific practice and zero (0) if otherwise. Specifically, socioeconomic, institutional, and biophysical factors were used as determinants of CSAPs adoption (Table 3).

**Table 3:** Descriptive statistics of the sampled households among smallholder farmers in Western Kenya.

Variable	Description	Mean	Std Dev.
Gender of the household head (hhh)	Dummy = 1 if male, 0 female	0.38	0.49
Education status of the household head (hhh)	Dummy = 1 if attained formal education, 0 otherwise	0.86	0.35
Age of the household head (hhh)	Age of the household head in years	51.91	13.74
Family size	Number of family members	5.78	2.91
Contact with extension agent	Dummy = 1 if yes, 0 otherwise	0.13	0.34
Access to weather information	Dummy = 1 yes, 0 otherwise	0.84	0.37
Arable land size	Total arable land size in acres	1.23	0.90
Owned livestock	Total livestock unit#	3.35	3.83
Perceived climate change	Dummy = 1 yes, 0 otherwise	0.96	0.19
Soil fertility	Dummy = 1 infertile, 0 fertile	0.24	0.43
Persistent soil erosion	Dummy = 1 yes, 0 otherwise	0.06	0.24

# Total livestock unit for cow, sheep, goat, and chicken calculated using a conversion of 0.7, 0.1,0.1, and 0.01 following Jahnke [47] and Musafiri et al. [26].

### 2.3 Sampling procedure and sample size

A cross-sectional survey and multi-stage sampling procedure in sampling the smallholder farmers was employed. First, Siaya County in Western Kenya was purposely selected at the first stage due to the high poverty levels, food insecurity, and climate-related shocks [17]. At the second stage, purposive sampling of two sub-counties, i.e., Alego-Usonga and Ugenya, from the six total sub-counties, including Bondo, Gem, Rarienda, and Ugunja in Siaya County was doen. This was informed by the climate risk dominance. A whole sampling procedure to collect data from the six and four wards in Alego-Usonga and Ugenya sub-counties was employed at the third stage. Proportionate to size sampling procedure was used in determining household heads sampled per ward. Finally, a random sampling procedure was used to collect data from 300 smallholder farming households in the two sub-counties. A total of 181 and 119 farmers were sampled from Alego-Usonga and Ugenya sub-counties. The population was 57, 553 and 33, 565 smallholder households in Alego-Usonga and Ugenya sub-counties [37]. Since a large population was involved, the Cochran formula was applied, arriving at a sample size of the 300 smallholders based on a 5% level of significance, and a 5.65% confidence interval, as described by Cochran [7].

### 2.4 Household interview

A semi-structured interview schedule for data collection was used. Before the actual data collection, the interview schedule was pre-tested using ten randomly selected smallholder farmers. Following feedback from the pre-testing, the interview schedule was modified/adjusted accordingly. The interview administration involved ten recruited and trained enumerators with close supervision by the authors. The interview schedule had questions on CSAPs adopted, smallholder farmers' socioeconomic, institutional, and biophysical variables. Smallholder farmers were requested to voluntary consent before participating in the study. The interview was administered to the household head.

### 2.5 Econometric framework

#### 2.5.1 Multivariate probit model

Smallholder farmers could adopt multiple CSAPs to improve food production and mitigate climate change impacts. To evaluate determinants of CSAPs adoption, interdependencies between error terms of different practices, including animal manure (M), agroforestry (A), soil water conservation (S), crop diversification (D), and crop-livestock integration (L) was assumed. Therefore, using a model that could estimate the determinants of practices simultaneously is imperative. A multivariate probit (MVP) model was used to assess the determinants of smallholders' simultaneous adoption of CSAPs. The MVP model estimates the determinants of simultaneous CSAPs adoption while the individual probit model considers one practice at a time [4]. The correlation of error terms where a positive sign represents complements or a negative sign indicates substitutes across different CSAPs [25, 35]. The MVP model can be presented in two systems equations. Following Kpadonou et al. [21], let U_a_ indicate the utility of adopting jth practice and U_n_ otherwise. Smallholders can adopt the jth approach if Yij = U_a_-U_o_>0. Therefore, net utility Y∗ij, a farmer obtain for adopting the jth practice, is a latent variable that can be predicted by the experimental factors and the multivariate normally distributed error terms (ε_i_) Eq. (1):

(1) $$Y_{ij}^{*}={\text{β}}_{j}X_{i}+{\text{ε}}_{i}$$

where X_i_ indicates a vector of independent variables, j climate-smart agriculture practice, $\beta_{j}$ Vector coefficient, and ε_i_ error term.

According to utility maximization theory, smallholder farmers could adopt CSAPs if the expected benefits are higher than non-adoption. This can be presented as an observable dichotomous outcome for each choice of CSAPs adopted by smallholder farmers could be described as shown in Eq. (2):

(2) $$Y_{ij}=\begin{array}{l} 1\;if\;Y_{ij}^{*}\\ 0\;otherwise \end{array}\;Where\;j=M,\;S,\;A,\;D,\;L$$

Where, $Y_{ij\;}$ Indicates a binary observable variable for adopting jth practice by the i^th^ farmer. Suppose adoption of CSAPs is assumed to co-occur; the error terms of the equation can be described using a variance-covariance matrix (Eq. (3)).

(3) $$\pi \;=\left(\begin{array}{lllll} 1 & \delta MS & \delta MA & \delta MD & \delta ML\\ \text{δSm} & 1 & \text{δSA} & \text{δSD} & \text{δSL}\\ \text{δAM} & \text{δAS} & 1 & \text{δAD} & \text{δAL}\\ \text{δDM} & \text{δDS} & \text{δDA} & 1 & \text{δDL}\\ \text{δLM} & \text{δLS} & \text{δLA} & \text{δLD} & 1 \end{array}\right)$$

Where rho (δ) is a pairwise correlation between any two CSAPs, the sign of δ between any two practices shows the relationship. As stated earlier, a positive sign represents complements, and a negative one indicates substitutes.

#### 2.5.2 Ordered probit model

From the MVP model, smallholder farmers adopt CSAP with higher utility than non-adoption. The MVP model considers smallholder farmers' adoption of specific CSAP conditional to other practices based on expected utility. The intensity of adoption is a count data that could be analyzed using Poisson regression. The Poisson regression is based on the assumption that all the events have the same probability of occurrence. However, the adoption intensity of CSAPs doesn't have the same chance of happening. The propensity of adopting the first CSAP could be different from the subsequent adoption of the practices (second to fifth) because smallholder farmers gain experience upon the first adoption. The smallholder farmers could have achieved better pay-off upon adopting the first practice and could be willing to adopt a combination of approaches to maximize the utility. Notably, the adoption of the practices could also differ based on their nature, including labor requirements, practical knowledge requirements, initial investments, and whether the benefits expected are in the short term or long term. However, smallholder farmers combine multiple CSAPs to increase the utility than those who adopt none, single, or few practices [21]. Adoption intensity (number of CSAPs adopted by the *i*^th^ farmer) was considered an as an ordinal variable that could be analyzed using the ordered probit model. The model allows for estimating determinants of ordinal variables (adoption intensity that 1, 2, 3, 4, and 5 CSAPs). The ordered outcome could be assessed as a latent variable Y∗, where Y∗ is the unobservable measure of smallholders' CSAPs adoption intensity [6, 35] as described in Eq. (4).

(4) $$Y_{j}^{*}=X_{j}^{\prime }\text{β}+{\text{u}}_{j}$$

For the *i*^*th*^ smallholder farmer where normalization is that the regressors x do not include and intercept, the adoption intensity increases with Y∗. The probability of observing a j outcome could be described by Eq. (5).

(5) $$\mathrm{Pr}\left(outcome\;i=j\right)=\text{Pr}(n_{j-1}<X_{j}^{\prime }\text{β}+{\text{u}}_{j}\leq \alpha_{j}$$

The coefficient β_1_, β_2_…  ​β_j-1_ were estimated jointly with the cut points α_1_, α_2_, …, α_j_ where j is the number of the possible outcomes. U_i_ is assumed to be normally distributed with a standard normal cumulative distribution function. The ordered probit model is pooled and works under the assumption that the unobserved heterogeneity is uncorrelated with the independent variables. Previous studies have adopted plot-level analysis to control unobserved heterogeneity that may affect the estimates using fixed or pseudo-fixed-effect models [21]. However, using plot-level analysis is not feasible in this study because of the nature of our data.

### 2.6 Ethical consideration

The study adhered to the research ethics guidelines recommended by the Board of Postgraduate Studies at the University of Embu. The processes involved undergoing a research ethics review and seeking informed consent from the participants. The study obtained informed consent from the interviewed farmers. The study ensured that the principle of anonymity and voluntary participation was respected. The enumerators were trained on seeking informed consent from the interviewees and agreement on voluntary participation in the study.

## 3 Results and discussions

### 3.1 Descriptive statistics of the smallholders

The descriptive characteristics of variables used in modeling are presented in Table 1 and Table 2. Smallholders' CSAPs adoption rate in Western Kenya is shown in Table 1. The adoption level of individual CSAPs ranged between 30% for agroforestry to 78% for crop diversification. The findings indicate that the adoption of individual CSAPs widely varies among smallholder farmers. The results were consistent with Ogada [15], who reported a varied adoption rate of agricultural practices in Western Kenya.

The adoption intensity of CSAPs ranged between zero to five (Table 2). Though some farmers (2.7%) adopted all the five CSAPs, a few farmers (2%) did not utilize any of the practices. Approximately 98% of the smallholder farmers practiced at least one CSAP. The findings agreed with Ehiakpor et al. [10], Kpadonou et al. [21], Ndiritu et al. [30], and Sileshi et al. [39], who reported high adoption rates of at least one CSAP. However, the adoption rates and intensity widely varied across the specific practice. The majority (80%) of the smallholder farmers adopted one to three CSAPs, and 15% implemented four out of the five practices. Only 2.7% of the sampled farmers adopted all the five CSAPs. Our findings implied a great potential to improve the adoption of agriculture practices for enhanced food and nutritional security, coping with climate change, reducing soil erosion, and uplifting economic gains among smallholder farmers. The simultaneous adoption of CSAPs needs to be interwoven with socioeconomic, institutional, and biophysical characteristics to improve society's welfare.

The socioeconomic, institutional, and biophysical variables displayed the profile of the sampled respondents (Table 3). The results showed that 38% of the sampled household heads were male and 68% female. These results implied that most of the farming population in Siaya County were female. Additionally, most (86%) of the sampled household heads were literate. The literacy level implied that most smallholder farmers in Western Kenya could effectively comprehend new agricultural innovations. Smallholders had an average age of 51.9 years. This is consistent with previous studies in Western Kenya Mutoko et al. [27] and Wetende et al. [46], who found the sampled households' heads were still in the active age bracket. However, the population was beyond the youths' frame of 35 years and below, implying that youths were not actively participating in agricultural production. Smallholder farmers had an average family size of 5.78 members, which is an essential variable that indicates farm labor availability.

Low access to extension agents of 13% was observed (Table 3). However, most sampled household heads received weather forecast information (86%) and perceived change in climate (96%). The smallholder farmers had small landholdings (1.23 acres) and tropical livestock units (3.35). Additionally, only a few household heads perceived their soil status as problematic, that is, 24% infertile soil and 6% persistent soil erosion.

### 3.2 The compliments and substitutes of climate-smart agricultural practices

The likelihood ratio test (chi^2^ = 658.201, p < 0.0001) of the error terms of different CSAPs equations from the MVP model was significant at a 1% level of significance, thus rejecting the null hypothesis that the equations were independent (Table 4). The results indicated that the equations for adopting individual CSAP were interdependent. Therefore, the alternative hypothesis of the interdependence between error terms of CSAPs was accepted. Thus, the justification for using the MVP model in analyzing the determinants of adopting the CSAPs. There were positive and negative correlation coefficients indicating both complements and substitutes between CSAPs. Our findings were similar to Ndiritu et al. [30], who reported complements and substitutes between sustainable intensification practices among smallholders in Kenya. Compliments between soil water conservation and animal manure, agroforestry and animal manure, crop diversification and soil water conservation, crop-livestock integration, and crop diversification were established. The complements of CSAPs could be attributed to the desire to improve agricultural productivity, adapt to climate change, and enhance income [35]. The CSAPs used as substitutes among the smallholder farmers included crop diversification and animal manure, crop-livestock integration and soil water conservation, crop-livestock integration, and agroforestry. Crop diversification and crop-livestock integration involve agricultural intensification. Farmers may find it less economical to combine farming revenues with animal manure, agroforestry, and soil water conservation to boost farming revenues.

**Table 4:** Correlation coefficients of the climate-smart agricultural practices (estimation from multivariate probit model).

CSA practice	Coefficient	Std. Err.	p value
Soil water conservation and animal manure (rho21)	0.127∗∗∗	0.095	0.008
Agroforestry and animal manure (rho31)	0.118∗∗	0.098	0.048
Crop diversification and animal manure (rho41)	-0.122∗∗∗	0.104	0.003
Crop-livestock integration and animal manure (rho51)	-0.087	0.092	0.435
Agroforestry and soil water conservation (rho32)	0.028	0.103	0.786
Crop diversification and soil water conservation (rho42)	0.474∗∗∗	0.090	<0.001
Crop-livestock integration and soil water conservation (rho52)	-0.124∗∗∗	0.096	0.001
Crop diversification and agroforestry (rho43)	0.044	0.107	0.682
Crop-livestock integration and agroforestry (rho53)	-0.173∗∗∗	0.089	0.001
Crop-livestock integration and crop diversification (rho54)	0.178∗∗∗	0.100	0.003

Likelihood ratio test of rho21 = rho31 = rho41 = rho51 = rho32 = rho42 = rho52 = rho43 = rho53 = rho54 = 0: chi^2^(10) = 125.5427 Prob > chi^2^ = 0.0001.

∗∗p < 0.05.

∗∗∗p < 0.01.

### 3.3 Determinants of climate-smart agriculture practices adoption

Factors that determined individual or simultaneous adoption of CSAPs were assessed. The Wald chi^2^ = 102.63, p = 0.0001 was significant (Table 5), justifying the plausibility of MVP analysis. Therefore, the null hypothesis that CSAPs such as animal manure, soil water conservation, agroforestry, crop diversification, and crop-livestock integration were independent was rejected. The results indicated that the practices were interdependent, and using the individual probit model produced biased estimates.

**Table 5:** Determinants of climate-smart agricultural practices adoption among smallholder farmers in Western Kenya.

Variable	Multivariate probit estimates					Individual probit estimates				
	M Coeff. (S.E)	S Coeff. (S.E)	A Coeff. (S.E)	D Coeff. (S.E)	L Coeff. (S.E)	M Coeff. (S.E)	S Coeff. (S.E)	A Coeff. (S.E)	D Coeff. (S.E)	L Coeff. (S.E)
Gender of the hhh	-0.172 (0.177)	0.031 (0.176)	-0.511∗∗∗ (0.182)	0.317 (0.192)	-0.248 (0.170)	-0.166 (0.177)	0.013 (0.177)	-0.502∗∗∗ (0.183)	0.361∗ (0.197)	-0.252 (0.170)
Education status hhh	0.555∗∗ (0.276)	-0.407 (0.279)	0.120 (0.276)	-0.431 (0.307)	0.086 (0.261)	0.549∗∗ (0.276)	-0.389 (0.279)	0.128 (0.276)	-0.428 (0.310)	0.089 (0.261)
Age of the hhht	0.012∗ (0.006)	0.000 (0.006)	-0.005 (0.007)	-0.003 (0.007)	0.005 (0.006)	0.012∗ (0.006)	0.000 (0.006)	-0.005 (0.007)	-0.003 (0.007)	0.005 (0.006)
Family size	-0.046 (0.030)	0.035 (0.029)	-0.056∗ (0.030)	0.043 (0.033)	0.001 (0.028)	-0.046 (0.030)	0.036 (0.030)	-0.060∗∗ (0.031)	0.042 (0.033)	0.002 (0.028)
Contact with extension agent	-0.440∗ (0.250)	0.715∗∗∗ (0.256)	-0.257 (0.246)	1.094∗∗∗ (0.351)	-0.194 (0.225)	-0.450∗ (0.251)	0.736∗∗∗ (0.257)	-0.251 (0.247)	1.147∗∗∗ (0.361)	-0.180 (0.225)
Access to weather information	-0.308 (0.215)	-0.269 (0.231)	0.467∗ (0.243)	-0.451∗ (0.267)	0.234 (0.216)	-0.311 (0.216)	-0.229 (0.230)	0.472∗ (0.246)	-0.477∗ (0.269)	0.224 (0.216)
Arable land	-0.041 (0.100)	-0.005 (0.096)	0.145 (0.098)	-0.096 (0.104)	0.171∗ (0.096)	-0.039 (0.099)	-0.010 (0.097)	0.150 (0.098)	-0.109 (0.104)	0.167∗ (0.095)
Livestock owned	-0.018 (0.022)	0.023 (0.021)	0.013 (0.021)	-0.014 (0.022)	0.051∗∗ (0.021)	-0.018 (0.022)	0.020 (0.021)	0.014 (0.021)	-0.011 (0.023)	0.051∗∗ (0.021)
Perceived climate change	0.838 (0.550)	-1.069∗ (0.581)	0.852 (0.562)	-4.631 (1.622)	0.440 (0.421)	0.834 (0.549)	-1.130∗ (0.587)	0.971∗ (0.570)	-	0.477 (0.418)
Soil fertility	0.515∗∗∗ (0.181)	-0.333∗ (0.184)	-0.013 (0.188)	-0.339∗ (0.195)	-0.223 (0.183)	0.512∗∗∗ (0.186)	-0.378∗∗ (0.190)	0.064 (0.194)	-0.274 (0.199)	-0.227 (0.184)
Persistent soil erosion	-0.210 (0.334)	1.429∗∗∗ (0.516)	0.534∗ (0.322)	0.123 (0.345)	-0.105 (0.326)	-0.223 (0.338)	1.351∗∗∗ (0.500)	0.586∗ (0.321)	0.080 (0.352)	-0.082 (0.324)
Constant	-1.741∗∗ (0.757)	1.698∗∗ (0.770)	-1.316∗ (0.794)	6.077 (2.623)	-1.300∗∗ (0.660)	-1.713∗∗ (0.755)	1.645∗∗ (0.774)	-1.303 (0.799)	1.699∗∗∗ (0.585)	-1.349∗∗ (0.659)

Number of observations = 300 Log likelihood = -848.359 Wald chi2 (56) = 102.63.

Prob > chi2 = 0.0001, ∗p < 0.1 ∗∗p < 0.05 ∗∗∗p < 0.01, robust standard error in parenthesis, M = Animal manure. S = Soil water conservation, A = Agroforestry, D = crop diversification, L = crop livestock integration.

The adoption of CSAPs is influenced by socioeconomic, institutional, farmer perceptions, and biophysical (Table 5). Household head's gender negatively affected agroforestry adoption. The finding suggests that females had a higher propensity to adopt agroforestry than males. The negative prediction was against the previous literature that male dominates farming resources and could be attributed to female empowerment [20, 35]. Given that the dominant cropping enterprise in the study area is sorghum, the increased adoption of agroforestry by female households could be attributed to the crop being referred to as a poor man's crop'. Our finding agreed with Kiptot and Franzel [20], who found that women highly practice agroforestry with crops of little or no commercial value. Smallholder farming in Western Kenya is women-dominated (Table 3). Most agricultural empowerment programs target women Diiro et al. [9], thus enhancing good farming practices among female farmers. The findings underscore the responsibility of women in climate change adaptation and sustainable agriculture.

The results revealed that the household head's education level positively determined animal manure adoption (Table 5). This implied that literate smallholder farmers had higher chances of applying animal manure in their farms than illiterate ones. The observation may be because the educated farmers may know the correct methods and amounts of animal manure application. The findings agreed with Kanyenji et al. [15] and Kassie et al. [16], who highlighted the importance of education in adopting animal manure. However, our results contradicted Oyetunde-Usman et al. [35], who found education determinant of organic manure adoption.

Household head's age positively predicted animal manure adoption. The findings suggested that the propensity to adopt animal manure increased with age. This could be attributed to the possibility that old farmers have evaluated the benefits of animal manure application over the long term. Further, the older farmers could have larger livestock herds than their young counterparts. The results corroborated with Oyetunde-Usman et al. [35], who found that adoption of animal manure was positively influenced by age. However, our findings contradict the hypothesis that old farmers are risk-sensitive and reluctant to adopt agricultural innovations [23, 26].

Family size negatively predicted agroforestry adoption. The findings implied that large families were less likely to adopt agroforestry. Family size is an important variable as it signifies the availability of labor to adopt an agricultural practice. The pessimistic prediction of family size on the adoption of agroforestry was unanticipated because the hypothesize was that large family sizes could be in a position to supply labor. After all, agroforestry is a labor-intensive technology. The results could be due to the probability of small family sizes using hired labor in implementing agricultural innovations. Our findings corroborated with Ehiakpor et al. [10] and Kpadonou et al. [21], who reported that family size negatively determines agricultural practices adoption. However, our findings disagreed with Bryan et al. [5], Kassie et al. [16], and Mwaura et al. [28], who found that family size positively influenced agricultural technologies utilization.

There was a significant positive influence of contact with extension agents on soil water conservation and crop diversification, while animal manure was negative. Extension help smallholder gains more insights into implementing agricultural technologies [49]. The findings could be linked to the need for technical know-how in implementing soil water conservation practices and crop diversification instead of animal manure, one of the traditional practices. The extension agents' contacts could have played a central role in equipping the farmers with practical skills of soil water conservation implementation and selecting crop diversification practices to improve agricultural productivity and adapt to climate change. Our finding agreed with Anang et al. [1] and Emmanuel et al. [11], who underscored extension services implication in enhancing agricultural interventions adoption.

Access to weather forecast information positively influenced the adoption of agroforestry and negatively affected crop diversification. The findings implied that receiving weather forecast information accelerated the propensity to adopt agroforestry while decreasing the likelihood of implementing crop diversification. The receipt of weather forecast information help smallholder farmers choose CSAPs for climate change mitigation. Our findings could be attributed to smallholders' need to implement long-term strategies for climate change adaptation, including agroforestry, instead of the short-term ones among smallholder farmers who received weather forecast information. Adopting agroforestry trees among smallholder farmers who received weather forecast information could be attributed to the multiple anticipated benefits, including improved soil carbon sequestration, food security, income, provision of shade and timber [36].

Arable land size exhibited a significant positive influence on adopting crop-livestock integration. Our findings suggested an increased likelihood of adopting crop-livestock integration with increased arable land size. The increased adoption of crop-livestock integration could be due to the need for larger farm sizes for livestock keeping and crop farming. The larger land could also grow fodder crops used as animal feeds. The result could be attributed to smallholder farmers apportioning their farms to different technologies with more extensive farm holdings. Our finding concurs with Darkwah et al. [8], Ehiakpor et al. [10], and Thinda et al. [42]. Notably, smallholder farmers with large landholdings benefit from the trade-off arising from crop-livestock integration, such as using the crop residue as animal feed and the livestock's application for soil fertility amelioration.

The TLU positively determined crop-livestock integration adoption. The findings suggested that the propensity of crop-livestock integration adoption increased with an increase in TLUs. The influence of TLU on crop-livestock integration could be attributed to the greater need for animal feeds among households with greater TLU, thus integrating crops and livestock to utilize the crop residues as animal feeds. Additionally, the manure produced from the livestock could also be incorporated into the agricultural land, thus enhancing soil fertility. Our findings were consistent with Kanyenji et al. [15] and Ndeke et al. [29], who found that TLU was a significant positive determinant of improved technologies adoption.

Farmers' perceptions of climate change positively explained soil water conservation adoption. The findings implied that household heads who perceived climate change had a higher likelihood of adopting soil water conservation practices. The increased adoption among smallholders who perceived climate change can be attributed to the anticipated reduction in food production. Therefore, smallholders' awareness of climate change could have motivated them to implement CSAPs. Our findings were in line with Joshi et al. [14] and Ochieng et al. [32]. However, smallholder farmers could fail to adopt sustainable agricultural practices even if they perceive climate change due to the high investment cost required [5].

Soil fertility significantly influenced animal manure adoption but negatively affected soil water conservation and crop diversification adoption. This implied that smallholders experiencing poor soil fertility had a higher likelihood of adopting animal manure and a lower propensity to utilize soil water conservation and crop diversification practices. The finding could improve soil fertility by using animal manure to increase income and food security. Further, the smallholder farmers can anticipate crop failure or lower yields from infertile plots, thus failing to implement high investment practices. Soil water conservation and crop diversification are not directly linked to soil fertility improvement. Therefore, smallholder farmers could find it suitable to implement animal manure for soil fertility amendment. Our findings were consistent with Fosu-Mensah et al. [12] and Mulwa et al. [25], who reported that smallholders with fertile plots were less likely to utilize agricultural innovations. This was attributed to reduced chances of crop failure in fertile fields.

Persistent soil erosion positively determined agroforestry and soil water conservation adoption. This suggested that smallholders who perceived continued soil erosion had a higher propensity to adopt agroforestry and soil water conservation practices. The findings could be applied in controlling soil erosion through agroforestry and soil water conservation structures. Agroforestry and soil water conservation practices reduce soil erosion and improve soil water retention, leading to higher crop yields and income [2, 40]. Additionally, smallholder farmers who perceived persistent soil erosion were more likely to experience crop failure, thus investing in CSAPs.

The discussion emphasized the MPV model results. The findings were compared with the individual probit model. Pretty similar estimates from individual and MVP models regarding coefficients, significance, and sign were observed. However, the MVP model was found to be more reliable than the individual probit as it explained the multiple CSAPs adoptions.

### 3.4 Determinants of climate-smart agriculture practices intensity

Adoption intensity is imperative among smallholder farmers to improve crop yields and income and mitigate climate change impacts [21.30.35]. Our results revealed that the LR Chi^2^ = 125.05, Prob > chi^2^ = 0.000 was significant, suggesting that the ordered probit model was credible.

The household head's gender negatively predicted CSAPs adoption intensity (Table 6). The results suggested that female-headed smallholders had a higher propensity to intensify agricultural practices than male-headed households. The findings contradict the notion that male-headed strengthen agricultural practices since they control production resources such as labor and land. The results conformed with the simultaneous adoption of CSAPs (Table 5). These findings could be useful to the women empowerment programs in the area [9]. Our results contradicted Oyetunde-Usman et al. [35], who reported that male-headed households intensified sustainable agricultural practices and attributed it to poor access to complementary inputs.

**Table 6:** Factors influencing the number of climate-smart agricultural practices adopted using an ordered probit model.

Variables	Coefficient	Std Error	p-value
Gender of the hhh	-0.340∗∗	0.144	0.018
Education status of hhh	-0.082	0.220	0.710
Age of the hhh	0.000	0.005	0.948
Family size	-0.007	0.023	0.752
Contact with extension agent	0.122	0.188	0.517
Access to weather information	0.184	0.180	0.308
Arable land size	0.142∗∗	0.078	0.068
Livestock owned	0.040∗∗	0.017	0.018
Perceived climate change	0.155	0.338	0.648
Soil fertility	-0.260∗	0.150	0.083
Persistent soil erosion	0.669∗∗∗	0.270	0.003

Number of observations = 300	LR Chi2 (11) = 125.05	Prob > chi2 = 0.000	
Log likelihood = -348.345	Pseudo R2 = 0.0347		

∗p < 0.1 ∗∗p < 0.05 ∗∗∗p < 0.01.

Arable land positively influences CSAPs' adoption intensity. The findings suggested that the propensity of adopting multiple CSAPs among smallholders increased with Arable land size. The results corroborated with section 3.3, thus highlighting the importance of landholding in agricultural intensification. On the other hand, livestock ownership significantly influenced CSAPs intensification, thus substantiating results reported in Table 5 and highlighting the importance of livestock in agricultural intensification. The observation is that livestock dropping was used as the source of manure. The findings align with Ehiakpor et al. [10], who established that livestock ownership significantly influenced sustainable agricultural practices adoption intensity. This was attributed to the probability of selling livestock to purchase farm inputs, including agrochemicals, fertilizers, and improved seeds.

The negative and significant prediction of soil fertility on adoption intensity implied that smallholder farmers who perceived infertile soil were less likely to intensify agricultural practices. Smallholder farmers under low soil fertility status are more likely to experience adverse effects of climate change, such as reduced crop yields. Poor soil fertility is a considerable drawback to agricultural production in SSA [19, 45]. Low soil fertility execrates the effects of climate change. Therefore, the smallholder farmers under deprived soil fertility intensify agricultural production to improve crop yields and lower crop failure risks. Notably, smallholder farmers experiencing good soil fertility anticipate fewer climate-related stocks, such as crop failure, thus intensifying their agricultural production [25].

Persistent soil erosion significantly influenced CSAPs adoption intensity, suggesting that smallholder farmers who perceived constant soil erosion had a higher propensity to intensify CSAPs. This is laudable because the smallholder farmers in erosion-prone areas could boost CSAPs adoption to reduce erosion compared with those in less erosion-prone areas. This is probably because CSAPs such as soil water conservation and agroforestry controls soil erosion. Hence, the joint adoption of CSAPs could reduce soil erosion prevalence, thus increasing crop yields and income. Therefore, the need to prioritize erosion control methods in agricultural fields to minimize [48].

## 4 Conclusions

The adoption level and intensity of CSAPs varied because of differences in the socioeconomic, institutional, and biophysical factors across sampled households. Positive and negative correlation coefficients between CSAPs were established, indicating that they acted as complements and substitutes. The critical determinants of multiple adoptions of CSAPs were household head's gender, education, age, family size, contact with extension agents, access to weather information, arable land, livestock owned, perceived climate change, infertile soil, and persistent soil erosion. Our findings revealed that gender of the respondent, arable land, livestock owned, soil fertility, and continued soil erosion were crucial determinants of CSAPs adoption intensity. Female-headed households, farmers' asset base, and farm factors influenced smallholder farmers' adaptive capacity.

Against the above background, it was recommended that policymakers design pro-farmers policies that promote the adoption of multiple agricultural practices to complement each other in mitigating the adverse impacts of climate change. Given that numerous factors determine the adoption of various CSAPs, policymakers should innovatively consider smallholders' perceptions of soil fertility, soil erosion, and climate change in optimizing CSAPs adoption. Therefore, the policymakers should target smallholder farmers who perceive poor soil fertility, high soil erosion, and climate change to enhance the adoption of CSAPs. In upscaling the adoption of CSAPs, governments and stakeholders should promote extension services and agricultural training for improved capacity building among smallholder farmers.

## Declarations

### Author contribution statement

Collins M. Musafiri; Milka Kiboi: Conceived and designed the experiments; Performed the experiments; Analyzed and interpreted the data; Wrote the paper.

Joseph Macharia: Conceived and designed the experiments; Performed the experiments; Wrote the paper.

Onesmus K. Ng'etich: Conceived and designed the experiments; Analyzed and interpreted the data; Wrote the paper.

David K. Kosgei; Betty Mulianga: Conceived and designed the experiments; Contributed reagents, materials, analysis tools or data; Wrote the paper.

Michael Okoti: Conceived and designed the experiments; Performed the experiments.

Felix K. Ngetich: Conceived and designed the experiments; Performed the experiments; Analyzed and interpreted the data; Contributed reagents, materials, analysis tools or data; Wrote the paper.

### Funding statement

This work was supported by 10.13039/100004421World Bank for the research grant through Kenya Climate-Smart Agriculture Project (KCSAP), a multi-disciplinary collaborative project titled "Validating Sustainable Land Management Technologies for Enhanced Carbon Sequestration and Improved Smallholder Farmer's Livelihoods."

### Data availability statement

Data included in article/supplementary material/referenced in article.

### Declaration of interests statement

The authors declare no conflict of interest.

### Additional information

No additional information is available for this paper.
